# Ten years of spasers and plasmonic nanolasers

**DOI:** 10.1038/s41377-020-0319-7

**Published:** 2020-05-25

**Authors:** Shaimaa I. Azzam, Alexander V. Kildishev, Ren-Min Ma, Cun-Zheng Ning, Rupert Oulton, Vladimir M. Shalaev, Mark I. Stockman, Jia-Lu Xu, Xiang Zhang

**Affiliations:** 1 School of Electrical & Computer Engineering and Birck Nanotechnology Center, Purdue University, West Lafayette, IN 47907 USA; 2 Purdue Quantum Science and Engineering Institute, Purdue University, West Lafayette, IN 47907 USA; 3 State Key Lab for Mesoscopic Physics and School of Physics, Peking University, Beijing, China; 4 Frontiers Science Center for Nano-optoelectronics & Collaborative Innovation Center of Quantum Matter, Beijing, China; 5 Department of Electronic Engineering and International Center for Nano-Optoelectronics, Tsinghua University, 100084 Beijing, China; 6 School of Electrical, Computer, and Energy Engineering, Arizona State University, Tempe, AZ 85287 USA; 7 The Blackett Laboratory, Imperial College London, South Kensington, London, SW7 2AZ UK; 8 Center for Nano-Optics (CeNO) and Department of Physics and Astronomy, Georgia State University, Atlanta, GA 30303 USA; 9 Nanoscale Science and Engineering Center, University of California, Berkeley, Berkeley, CA 94720 USA; 10 Faculties of Sciences and Engineering, University of Hong Kong, Hong Kong, China

**Keywords:** Lasers, LEDs and light sources, Optical materials and structures, Nanophotonics and plasmonics

## Abstract

Ten years ago, three teams experimentally demonstrated the first spasers, or plasmonic nanolasers, after the spaser concept was first proposed theoretically in 2003. An overview of the significant progress achieved over the last 10 years is presented here, together with the original context of and motivations for this research. After a general introduction, we first summarize the fundamental properties of spasers and discuss the major motivations that led to the first demonstrations of spasers and nanolasers. This is followed by an overview of crucial technological progress, including lasing threshold reduction, dynamic modulation, room-temperature operation, electrical injection, the control and improvement of spasers, the array operation of spasers, and selected applications of single-particle spasers. Research prospects are presented in relation to several directions of development, including further miniaturization, the relationship with Bose–Einstein condensation, novel spaser-based interconnects, and other features of spasers and plasmonic lasers that have yet to be realized or challenges that are still to be overcome.

## Plasmonic nanolasers and Spasers: Their evolution, properties, and future applications

A review of plasmonic nanolasers charts breakthroughs in the technology over the past decade and points towards future research pathways and potential new applications. Plasmonic nanolasers, or spasers, are the counterparts of lasers, but instead of emitting photons, spasers emit composite particles made of photons and plasmons on the surfaces of metal nanoparticles. Their applications range from spectroscopic detection, on-chip light sources, and microscopy to optical sensors and probes. Now, an international team of researchers, led by Cun-Zheng Ning from Tsinghua University, has conducted a comprehensive review of the evolution spasers, from their first experimental demonstrations through to technological advances in the field and future research and new applications. After showing how the drive for miniaturization led to their creation, the review then summarises their properties and crucial progress made, and offers perspectives on unresolved issues and future challenges in the field.

## Introduction

Among the discoveries and inventions that have defined science, technology and, generally, civilization as we know it, the invention of the laser 60 years ago stands out^1–3^. Lasers provide the unique capability to concentrate energy in the form of coherent radiation in the smallest phase-space volume possible in optics. This allows the formation of coherent beams with a minimum angular divergence or the focusing of radiation to the smallest possible spots, with sizes down to a half-wavelength. Lasers also allow the concentration of optical energy in the time domain to the shortest possible pulses, with a duration on the order of a single optical cycle, providing access to sub-cycle phenomena with durations on the order of 100 attoseconds^4,5^.

Historically, lasers were heralded for their monochromaticity, high intensity, and low beam divergence; however, today, stimulated emission is utilized as a tool that offers exquisite control to engineer light fields with well-defined frequencies, statistical properties, polarizations, and spatial profiles. The range of applications is vast. Miniaturization has always been a persistent topic of research in photonics; just 2 years after Maiman’s first laser^3^, semiconductor lasers^6^ emerged, which were orders of magnitude smaller. Technologically, semiconductor lasers are naturally more compact, but with the advent of heterostructures^7^, they also became able to operate at lower power under electrical injection, even under battery power. As transistor scaling and integration set in motion the microelectronics and computer revolutions, the integration of microelectronics with photonics was long perceived to be unavoidable^8^. When the smallest dimensions of lasers eventually reached wavelength scales in the 1990s (see, e.g., ref. ^9^), they were still several orders of magnitude larger than transistors. However, it was realized that micro- and nanoscale optical resonators could be utilized to control spontaneous emission. From this paradigm, spontaneous emission control emerged as a modern topic of research in the field of nanolasers.

By the turn of the millennium, optically pumped photonic crystal (PC) nanolasers were among the smallest devices^10^. Other forms of small lasers soon followed starting in the early 2000s^11^, such as nanowire lasers^12–16^. However, the quest to reduce the resonator size made it extremely difficult to produce such small PC lasers operating under electrical injection. Metal contacts that closely approached the cavity inevitably introduced both scattering and absorption losses. Complications of poor heat conduction and low mechanical stability also arose as these devices were constructed from thin suspended membranes of semiconductor material. Meanwhile, many studies on metallic waveguides were indicating the possibility of confinement beyond the wavelength range of light^17–23^. As such approaches introduced metallic loss, integrated gain strategies were also devised to prolong the propagation of light^24–26^. The final ingredient, and the key to effectively scaling down the physical size of lasers, was the emergence of metal-based cavities operating in dielectric modes^27^ or plasmonic modes in 2007^28^. Such devices embraced the capability of metals to provide all of the requirements for any laser^29–32^: optical confinement, feedback, electrical contacts, and thermal management^33–36^. Such approaches relied on metals to support surface electromagnetic waves that used electron oscillations to promote optical confinement. It was Bergman and Stockman^33^ who first realized in 2003 that these surface plasmon waves could also be amplified by stimulated emission. Thus, the concept of the spaser (an acronym for surface plasmon amplification by stimulated emission of radiation) was born. This realization provoked considerable interest due to the predicted ability of such a new device to generate coherent plasmonic fields, along with the prospective applications of this capability.

**Fig. 1:**
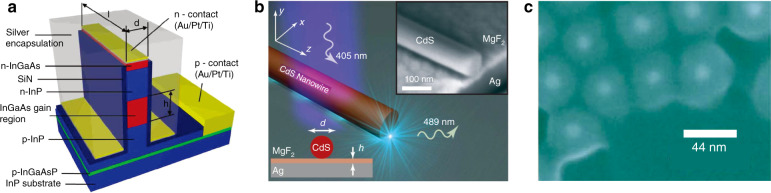
The first reported plasmonic nanolasers, or spasers, in 2009. **a** Nanolaser based on a metal-insulator-semiconductor-insulator-metal plasmonic gap mode, where vertical confinement was achieved by means of a double heterostructure^34^. **b** Plasmonic nanolaser based on a two-dimensionally confined nanowire plasmonic mode^35^. **c** Spaser based on a three-dimensionally confined metal nanoparticle mode^36^.

**Fig. 2:**
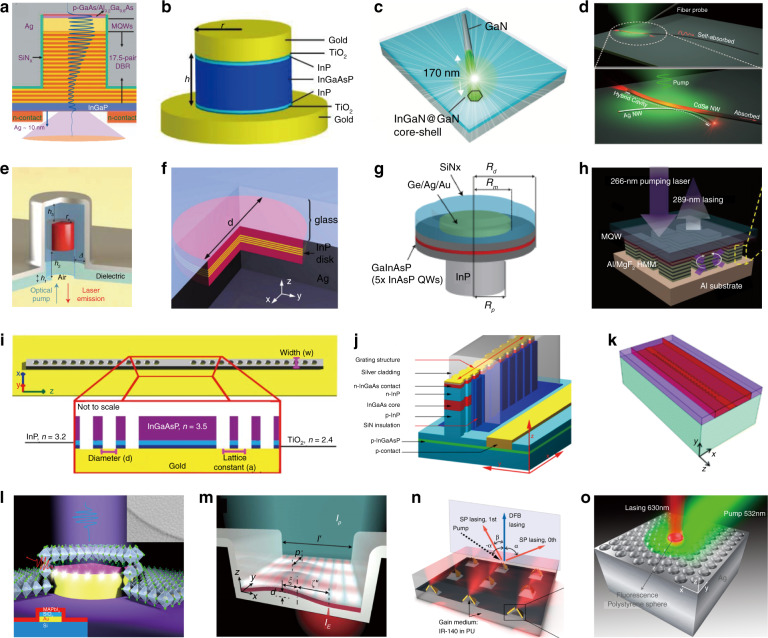
Zoo of nanolasers designed since the first demonstrations of plasmonic nanolasers in 2009. **a** A cavity combining distributed Bragg reflectors with metal^56^. **b** A double-patch metal cavity^57^. **c** A core-shell nanowire on a single-crystal silver film^46^. **d** A CdSe nanowire tangentially coupled to a silver wire^58^. **e** A semiconductor-metal co-axial cavity^59^. **f** quantum wells in a metal pan^60^. **g** A microdisk laser with a plasmonic disk^32^. **h** A metamaterial laser^61^. **i**–**k** 1D plasmonic crystal lasers^51,62,63^. **l** A perovskite-based plasmonic nanolaser^52^. **m** A metallic trench Fabry–Pérot resonator plasmonic nanolaser^53^. **n**, **o** 2D plasmonic crystal nanolasers^54,55^.

Ten years ago, three teams^34–36^ independently demonstrated the first plasmonic nanolasers, or spasers, from different perspectives and with different motivations (Fig. 1). These plasmonic nanolasers are extremely compact coherent light sources with ultrafast dynamics and a broad palette of promising applications^8,37^. The original spaser design was a nanoshell-based localized surface plasmon (LSP) spaser^36^ containing a metal nanosphere as the plasmonic core, surrounded by a dielectric shell containing a gain material, typically dye molecules. Since then, other nanoshell LSP spasers have been reported^38,39^. Such spasers are the smallest coherent generators produced so far, with sizes on the order of several to tens of nanometers. On the other hand, devices that were originally termed plasmonic nanolasers^34,35^ are based on semiconductor-metal plasmonic gap modes with a surface plasmon polariton (SPP) mode propagating in one of the dimensions. In terms of the physics principles at work, these SPP nanolasers^34,35^ are the same as spasers^36^, with the only difference being whether localized^36^ or propagating plasmon modes^34,35^ are involved. Therefore, we will not intentionally distinguish in this paper between SPP nanolasers and LSP spasers, and we use the two terms interchangeably. Later, this type of nanolaser (or SPP spaser) was widely developed and perfected^40–45^. There are also LSP nanospasers that are similar in design to SPP nanolasers but are true nanospasers whose dimensions are all on the nanoscale. Such a spaser consists of a semiconductor nanorod as a gain material deposited on top of a single-crystal nanofilm of a plasmonic metal^46^. These spasers possess very low thresholds and have been demonstrated for wavelengths spanning the visible spectrum by changing the semiconductor composition while keeping the geometry fixed^47–49^. A similar SPP spaser has also been demonstrated with quantum-dot gain media^50^. The great progress achieved over the last decade has led to rapid evolution from the very preliminary proof-of-concept demonstrations to a great variety of plasmonic nanolaser designs^32,46,51–63^ (Fig. 2), including more realistic devices aimed at specific applications. For example, their intrinsic capabilities suggest their potential for application in optical interconnects^8,41,64–70^, near-field spectroscopy and sensing^42,44,53,71,72^, optical probing for biological systems^38,73^, and far-field beam synthesis through near-field eigenmode engineering^55,74–79^. Although critical design problems still challenge the research community, there are also unprecedented opportunities for new advances in enhancing gain^8,80^ and plasmonic materials, artificial intelligence (AI)-driven optimal designs^81^, and fabrication protocols^80^ that would enable new, extremely compact devices with ultrafast operation speeds.

This review article provides a historical overview of spaser and plasmonic nanolaser research over the past decade. We aim to place recent breakthroughs into the context of the original historical motivations for laser miniaturization. This review article does not attempt to focus on the applications of nanolasers, which have recently been covered elsewhere^8,37^. Rather, it is a commentary on how we can exploit the control that laser miniaturization offers. The purpose of this review is thus to identify potential future research directions that build on our current perspective. For example, nanolasers naturally offer the highest available degree of control over both spontaneous and stimulated emission processes. Some interesting questions arise: If we are able to effectively control spontaneous emission into a single mode, would such an LED be sufficient without driving above the threshold into lasing? How small can a nanolaser be? Are the smallest nanolasers necessary, or is there an optimal length scale to ensure that other attributes meet essential application requirements, such as the energy efficiency and signal-to-noise ratio^8,69^?

## Fundamental properties of spasers

**Fig. 3:**
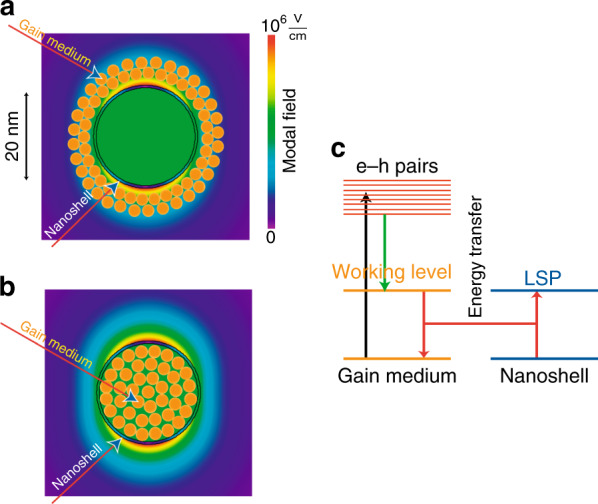
Conceptual schematic of a realistic spaser geometry and composition as well as its action principle^82^. **a** Schematic of the geometry and composition of a nanoshell spaser surrounded by a gain medium. The local fields (per one plasmon in the dipolar spasing mode) are shown with the color scale defined by the bar to the right. The plasmonic nanoshell and gain medium (orange dots) are indicated. **b** The same as **a** but for the gain medium inside the shell. **c** Schematic of spaser operation. The energy levels of the gain medium are depicted to the left, and those of the plasmonic core (the nanoshell, in this case) are shown to the right. An external source (optical or electrical, indicated by a vertical black arrow) injects electrons into the conduction band, creating a non-equilibrium (hot) electron-hole plasma. The hot carriers relax to the bandgap (vertical green arrow), possibly forming excitons. These excitations decay radiationlessly, transferring their energy to SPs of the nanoshell (shown by coupled red arrows). These SPs stimulate the emission of more SPs, causing an avalanche of generation that is eventually stabilized by the saturation of the gain medium.

A schematic of the geometry and fundamental operating principles of a spaser is presented in Fig. 3. The gain medium depicted in Fig. 3 can be either a nanoshell consisting of a bulk semiconductor, semiconductor (colloidal) quantum dots (QDs), or dye molecules. For concreteness, we will describe the operation of a spaser in terms of a bulk semiconductor nanoshell; the cases of QDs and dye molecules are quite similar.

The external source pumps electrons into the conduction band (CB) and holes into the valence band (VB) of the gain medium, creating a population inversion. These carriers relax, creating an inversion at the bandgap (the creation of excitons appears to be unlikely due to the high required free carrier density). The CB ↔ VB transitions in the gain medium are coupled to the LSP excitation/annihilation transition (represented in Fig. 3c by the coupled red arrows) by near-field radiationless transitions induced by a coupling term in the Hamiltonian H′ = −**dE**_*n*_ where **d** is an electronic transition dipole in the gain medium.

A semi-classical theory of spasers, in which the gain medium is treated quantum mechanically and the SPs are treated classically, has been presented in ref. ^82^. A nanoscale spaser (nanospaser) has many unique and general properties due to its nature as a deeply subwavelength, quasistatic device. Note that in this case, no electromagnetic scale (wavelength, skin depth, decay length, etc.) is relevant, and spaser theory scales with the only important scale: the size of the metal nanosystem, which we will denote by *R*. Consequently, a spaser possesses a series of general properties, some of which make it uniquely different from micro- or macroscopic lasers.

(i) The condition for spasing does not depend on the spaser size and is given by the following inequality^83^:
  1 $$\frac{4\pi}{3}\frac{{\vert{{\mathbf{d}}}\vert}^2{n_e}Q}{\hbar {\Gamma_{12}}\varepsilon_d}\,\ge\,1$$
  where *n*_*e*_ is the volume density of active electrons in the gain medium, the metal quality factor is defined as $Q = \left| {\varepsilon _{\mathrm{m}}} \right|/{\mathrm{Im}}\varepsilon _{\mathrm{m}}$, *ε*_m_ is the permittivity of the metal in the spaser core at the spasing frequency, Γ_12_ is the spectral width of the spasing transition, and *ε*_d_ is the permittivity of the surrounding dielectric medium. This condition can be recast in a very simple form^83^ in terms of the gain, *g*, that the spaser gain medium must possess in the bulk, *g* ≥ *g*_th_, where the threshold gain is *g*_th_ = *k*/*Q*, with $k = \omega \sqrt {\varepsilon _d} /c$ being the radiation wave vector in the surrounding medium. For a good plasmonic metal of *Q* ~ 10–100, this spasing condition is very realistic and easy to satisfy with semiconductor or dye-gain media. Because the spasing condition (1) does not depend on the spaser size, the spaser can be made truly nanoscopic.
  There are several experimentally demonstrated spasers, all of them possessing a shell geometry similar to that in Fig. 3a, whose sizes are truly nanometric (from several nanometers to several tens of nanometers)^36,38,39^. The unique applications of these nanospasers enabled by their nanometer-scale sizes include their biomedical use as intracellular labels and theragnostic agents. Such applications include cancer diagnostics and therapeutics (theragnostics)^38^ and super-resolution (stimulated emission depletion, or STED) imaging^39^.
(ii) The spasing frequency, *ω*_s_, is a linear average of the gain transition frequency, *ω*_21_, and the LSP frequency, *ω*_p_, namely,
  2 $$\omega _{\mathrm{s}} = \frac{{\gamma _{\mathrm{p}}\omega _{21} + \Gamma _{12}\omega _{\mathrm{p}}}}{{\gamma _{\mathrm{p}} + \Gamma _{12}}}$$
  where *γ*_p_ is the LSP resonance width. This is identical to the frequency pulling effect in conventional lasers with detuning^84^, except that the cavity frequency is replaced by the plasmon frequency. The spasing frequency does not depend on the spaser size or on the pumping rate. It is generally different from both the gain-medium transition frequency, *ω*_21_, and the LSP frequency, *ω*_p_. The frequency walk-off effect is inherent to both lasers and spasers, but in the latter case, it can be very significant due to the large spectral widths involved. One of the consequences of this fact is the possibility of tuning the spasing frequency throughout the entire visible range by changing the bandgap of the semiconductor gain medium while leaving the geometry and composition of the metal core unchanged^47^.
  The fact that the nanospaser frequency (2) depends only on the spaser shape and material composition but not on its size makes a nanospaser an outstanding frequency-based sensor of its dielectric environment. It is also uniquely sensitive to an extremely small amount of analyte due to its nanoscopic modal volume.
(iii) For a nanospaser, the spontaneous decay rate, *γ*_2_, of the gain-medium excitation into the LSP spasing mode is plasmonically enhanced and can be expressed for a nanoshell spaser^82^ as follows:
  3 $$\gamma _2 = \frac{{2|{\mathbf{d}}|^2}}{{\hbar \varepsilon _{\mathrm{d}}\varepsilon _{\mathrm{m}}^\prime \varepsilon _{\mathrm{m}^\prime}^\prime R^3}}$$
  where $\varepsilon _{\mathrm{m}}^\prime = {\mathrm{Re}}\varepsilon _{\mathrm{m}}$, $\varepsilon _{\mathrm{m}^\prime}^\prime = I{{m}}\varepsilon _{\mathrm{m}}$, and *R* is the nanoshell radius. An estimate with silver as a metal in the red spectral region and realistic parameters, *d* = 15 D and *R* = 5 nm, yields *γ*_2_ ≈ 5ps^−1^ ^82^. This rate exceeds the rates of other decay channels in the gain medium. This implies that the injection of a carrier into the CB of the gain medium leads to a spontaneous emission of an SP in the metal core, and as a result, *N*_p_ ∝ *I*_p_, where *N*_p_ is the SP population of the spasing mode and *I*_p_ is the rate of pumping. In such a case, the rate of radiation of the spaser, *I*_*r*_, as a function of the pumping rate (the so-called L–L curve) is linear even for a very small *I*_p_, far below the spasing threshold. Above the spasing threshold, the emission rate is increased by a factor of *N*_p_, and the L–L curve is always a straight line as a function of *I*_p_. Thus, the spasing threshold is imperceptible in the L–L curve. Because *γ*_2_ ∝ *R*^−3^, as seen in Eq. (3), such an “ultralow threshold” or “thresholdless” behavior always occurs for sufficiently small nanospasers. Such behavior has been demonstrated in a number of experiments; see, e.g., ref. ^47^.
(iv) Above the spasing threshold, SP emission is stimulated, and its emission rate can be estimated to increase with the pumping intensity *I*_p_ as $\gamma _2^{\left( {{\mathrm{st}}} \right)}{\mathrm{\sim }}\gamma _2N_{\mathrm{p}} \propto I_{\mathrm{p}}$. Because *N*_p_ scales as ∝*R*^3^, the generated spaser relaxation rate, $\left( {\gamma _2^{\left( {{\mathrm{st}}} \right)}} \right)^{ - 1}$, does not depend on *R* and is proportional to the pumping rate. Hence, a nanospaser can be used for direct modulation with a maximum frequency of ~5 THz that increases with pumping; it can also be used to generate ultrafast pulses with a duration of <100 fs (see the theory in ref. ^82^). Experimental evidence indicates that the direct modulation rate of a spaser can exceed 1 THz and that the pulses generated can be shorter than 0.8 ps^85^.
(v) The absence of a perceptible threshold in the L–L line, *I*_r_(*I*_p_) or *N*_p_(*I*_p_), does not imply that there actually is no threshold for coherent generation, as has been discussed elsewhere^86,87^. A finite threshold still exists, and it can be determined from the second-order autocorrelation function of the spaser radiation, *g*^(2)^(*τ*). This function is conventionally defined as

4 $$g^{(2)}\left( \tau \right) = \frac{{\left\langle {P\left( {t + \tau } \right)P\left( t \right)} \right\rangle }}{{\left\langle {P\left( t \right)} \right\rangle ^2}}$$

where *P*(*t*) is the probability of detecting a photon from the spaser at time *t* and *τ* is the time delay between the detection of two consecutive photons. This function is normalized such that *g*^(2)^(*τ*) → 1 for *τ* → ∞. Such higher-order correlation functions are conventionally used to characterize the statistics of photons emitted by various sources^84,88^.

Below the spasing threshold, the local and emitted fields of a spaser possess random statistics. In the extreme limit of Gaussian statistics, *g*^(2)^(*τ*) → 2 for *τ* → 0. Generally, below the threshold, one expects 2 ≥ *g*^(2)^(0) > 1. Immediately above the threshold, the spaser generation statistics change, and for pumping intensities at this threshold and higher, we have *g*^(2)^(*τ*) → 1 for all *τ*. Such threshold behavior is characteristic of lasers generating emission in a single mode^88^. For a nanospaser, this behavior has been convincingly demonstrated in experiments^46,47^. Note that such behavior corresponds to a non-equilibrium phase transition above which the local fields around the spaser become coherent and large, in contrast to the case of spontaneous SP emission.

## Physical motivations for small lasers

### Background of spaser invention

Even with all the remarkable progress in laser technology achieved over the last 60 years, the spatial concentration of laser radiation is still limited to a half-wavelength, i.e., a distance of ~1 μm, due to the wave nature of optical radiation. However, there are a large range of objects and phenomena at the nanoscale that represent a realm of significant fundamental and applied interest, as envisioned by Feynman^89^. Among them, natural objects such as subcellular structures and biological macromolecules, with sizes on the order of 10 nm, and engineering structures such as transistors, with sizes even below 10 nm, stand out. Therefore, coherent sources at corresponding optical frequencies have great fundamental and applied significance.

Obviously, photons, which are the quanta of electromagnetic fields, cannot be compressed to sizes much less than the diffraction limit, which is on the order of the wavelength, $\frac{{2\pi c}}{{\omega \sqrt \varepsilon }}$, where *c* is the speed of light in vacuum, *ω* is the optical frequency, and *ε* is the permittivity of the medium. This size, even for highly polarizable dielectrics with *ε* ~ 10, is still on the order of hundreds of nanometers, being one or more orders of magnitude greater than our target size of 10 nm. The use of plasmonic nanoparticles and pointed probes to concentrate optical energy due to plasmonic resonance enhancement and geometric concentration is a successful approach for nanoscopy, nanospectroscopy, and nanoscale detection and sensing^90–93^.

Despite all this progress, a coherent nanoscale optical source is still highly desirable and promising. The idea of such a nanosource is that the photons in a laser are, in a sense, coincidental. The general properties that a field’s quanta must satisfy to be suitable for coherent quantum generation, which photons do satisfy, are as follows: (i) bosonic statistics, which are needed to coherently accumulate a significant number of such quanta in a single state (mode); (ii) high linearity or harmonicity, which provides the possibility of accumulating such quanta and achieving a high field amplitude without a significant nonlinear (anharmonic) frequency shift (“chirp”) or deterioration of the quality factor of the resonance; and (iii) electroneutrality, which is necessary because otherwise, the accumulation of *N* quanta would cause an increase in their Coulomb energy scaling as ∝*N*, which would be unsustainable for *N* ≫ 1.

These properties are all satisfied by surface plasmons (SPs)—both LSPs and SPPs^92^. LSPs are fundamentally, by far, the most strongly localized optical-frequency quanta^94^. The reason is that the modal field potential of an LSP, *φ*_*n*_(**r**), satisfies the quasistatic equation

5 $$\frac{\partial }{{\partial {\mathbf{r}}}}\theta \left( {\mathbf{r}} \right)\frac{\partial }{{\partial {\mathbf{r}}}}\varphi _n\left( {\mathbf{r}} \right) - s_n\frac{{\partial ^2}}{{\partial {\mathbf{r}}^2}}\varphi _n\left( {\mathbf{r}} \right) = 0$$

Here, we assume that the system consists of two components—a metal and a dielectric—and that *θ*(**r**) is a characteristic function that is equal to 1 in the metal and 0 in the dielectric^94^; 1 > *s*_*n*_ > 0 is an eigenvalue, and *φ*_*n*_(**r**) is the corresponding eigenfunction; and homogeneous Dirichlet–Neumann boundary conditions hold. This equation does not possess any spatial scale except that defined by *θ*(**r**), implying that the LSPs are localized at the minimum scale of the metal nanoparticles constituting the system, which is assumed to be nanometric.

The LSPs defined by Eq. (5) obviously satisfy the three requirements formulated above: (i) Their modal field is a vector, ${\mathbf{E}}_n\left( {\mathbf{r}} \right) = - \frac{\partial }{{\partial {\mathbf{r}}}}\varphi _n\left( {\mathbf{r}} \right)$; consequently, they are of spin 1 and are bosons, in accordance with the Pauli theorem^95^. (ii) They are electroneutral, as follows from Eq. (5). (iii) LSPs are known to be highly harmonic due to their nature as collective excitations of a large number of electrons. The same properties are true of SPPs, except that they are propagating waves and, consequently, delocalized compared to LSPs. In addition, SPs interact with charges via their modal electric fields **E**_*n*_(**r**) just as photons do. Consequently, SPs can potentially be used instead of photons for quantum amplification and generation. A fundamental advantage of using SPs in lieu of photons is that they are nanoscopic by their localization scale. This idea of nanoscale spaser has been introduced in ref. ^33^; see also refs. ^82,96–99^.

### Threshold control in nanolasers

One of the original motivations for laser miniaturization was to reduce power consumption, which can be realized by simply reducing the physical volume of the gain material^69^. In the 1990s, Purcell’s effect was extensively explored to control the spontaneous emission rate and, thereby, the spontaneous emission coupling factor(*β*)^100–102^ through various methods of laser size reduction. A laser’s photon production, by means of either optical or electrical pumping, is shared between the available optical modes—the more optical modes there are, the higher the pump rate required to achieve the lasing threshold. The advantage thus comes from reducing the number of optical modes, *N*_m_, by using small optical cavities: this simply enables a higher proportion of spontaneous emission to seed the laser modes. To a first-order approximation, under the assumption that each optical mode competes equally for emission, the fraction of spontaneous emission, *β*, into a single laser mode is $\beta \sim N_{\mathrm{m}}^{ - 1}$. This concept inspired intense research aimed at creating the smallest possible lasers to achieve “thresholdless lasers” emitting almost no spontaneous emission^103^.

Because the stimulated emission needs to dominate in the lasing state, the lasing threshold can sometimes be defined intuitively as the condition in which the rates of spontaneous and stimulated emission into the laser mode are equal^37^. According to Einstein’s 1916 paper on spontaneous and stimulated emission coefficients, this condition is equivalent to saying that a lasing mode should contain one photon to reach the threshold^104^. Hence, for a given cavity loss rate, *γ*, the minimum power needed to maintain a single photon in the lasing mode is $\gamma \times h\upsilon \times \frac{1}{\beta }$, where *hυ* is the energy of that single photon. The total threshold power should also include the power needed to pump and maintain the carriers at the upper energy level for population inversion. The minimum power needed to maintain population inversion is *γ*_SP_ × E_21_ × *V* × *n*_tr_, where *γ*_SP_ is the spontaneous emission rate, *E*_21_ is the energy required to pump carriers into the excited energy level (~*hυ*), and *V* and *n*_tr_ are the physical volume and transparency carrier density, respectively, of the gain material.

An analysis of the rate equations yields the same physical picture, from which we can obtain the following normalized threshold pump rate, *R*_th_, for either photons or electrons^37^:

6 $$R_{{\mathrm{th}}} = \gamma \left( {1 + \beta ^{ - 1}} \right)\left( {1 + \zeta ^{ - 1}} \right)/2$$

where *ζ* = *γ*/*α*_*g*_, with *α*_g_ being the loss due to the gain material. There is a useful symmetry to this threshold definition: $\beta \propto N_{\mathrm{m}}^{ - 1}$ quantifies how the pump energy is divided amongst a device’s optical modes, and *ζ* ∝ (*V* × *n*_tr_)^−1^ quantifies the energy overhead for achieving population inversion. Note that *ζ* is unbounded—for example, the operation of a laser is typically dominated by cavity loss (ζ → ∞) for a “4-level” gain material. The threshold is minimized in cases where *β* → 1 and *ζ* → ∞, such that *R*_th_ → *γ*. Here, the minimum pump rate is exactly the rate at which photons are being lost from the cavity mode. Under this definition, thresholdless lasing is not physically possible.

### Accelerated laser dynamics

One of the important features of plasmonic lasers is their potential for ultrafast modulation. The spatial and spectral localization of nanocavity modes leads to modified dynamics due to the enhanced interactions between the gain medium and the cavity mode, i.e., the same Purcell effect^105^ mentioned above. The Purcell effect changes the natural spontaneous emission lifetime, ***τ***_0_, by the Purcell factor, *F* = ***τ***_0_/***τ***, where ***τ*** is the modified lifetime. The accelerated spontaneous emission into lasing modes enables nanolasers to have ultrafast responses^106^. Theoretical studies and numerical simulations have shown that nanolasers can be modulated at 100s GHz or even up to THz^107–113^. Recently, a nanolaser emitting sub-picosecond pulses has been experimentally demonstrated^85^.

Beyond the Purcell factor, accelerated laser dynamics modify the interactions between the gain material and the available optical modes. Some optical modes may couple preferentially to the gain material, while others could be suppressed. For the *n*th optical mode, the corresponding modal spontaneous emission lifetime is ***τ***_*n*_. The total Purcell factor is a sum over the modal Purcell factors, $F = \tau _0\tau ^{ - 1} = {\sum \nolimits_n} {\tau _0\tau _n^{ - 1}} ={\sum \nolimits_n} {F_n}$. The effect on stimulated emission is two-fold: first, all light-matter interactions within the gain medium are accelerated, including stimulated emission; second, the modal spontaneous emission factors are modified, such that for the *n*th mode, *β*_*n*_ = *F*_*n*_/*F*. This, in turn, modifies the lasing threshold for a particular mode. An increased *β*-factor further accelerates the temporal response under direct laser modulation^69,82,107–113^. Accessing high Purcell factors through high-cavity quality is not valid when the cavity resonance is narrower than the inhomogeneously broadened emission linewidth. Furthermore, it is not necessarily advantageous for laser performance. For example, long photon lifetimes limit the modulation bandwidth of a laser^108^.

One of the related issues with short carrier lifetimes is the validity of the rate equation approximations^113^. The complete set of Maxwell–Bloch equations describing a semiconductor laser includes coupled rate equations for photons and the population as well as the medium polarization equation. Due to the typically short nature of the polarization lifetime (sub-ps) compared to the carrier lifetime (~1 ns), only the photon and population rate equations remain, whereas the polarization equation may be adiabatically eliminated. For a plasmonic nanolaser, however, this approximation becomes questionable due to the greatly enhanced spontaneous emission rate, which could make the carrier lifetime more similar to the polarization lifetime. Therefore, the ultrafast modulation of plasmonic nanolasers, in general, cannot be analyzed based on the conventional rate equations alone. The validity of the rate equations was recently studied^113^ together with an analysis of the modulation of plasmonic nanolasers using a more complete set of equations beyond the rate equation approximation. Although there have been extensive theoretical studies, a systematic experimental investigation of plasmonic nanolasers is still lacking, with only a limited number of papers having been published on this topic. The operation of plasmonic nanolasers under direct current modulation has not been demonstrated. Many theoretical predictions have not been experimentally validated. Other aspects of modulation related to size and energy efficiency have been discussed more extensively elsewhere^69^.

### Loss and gain in plasmonic nanolasers

One of the important issues for plasmonic nanolasers is to overcome the metal loss by means of optical gain. Since it is known that the typical semiconductor gain is on the order of 10^3^–10^4^ cm^−1^ (the material gain), whereas the absorption of a metal can be as large as 10^6^ cm^−^^1^, it seems to be impossible to ever overcome the metal absorption when an SPP mode is propagating at a semiconductor-metal interface, where the mode exists partially on both sides. One of the interesting features of the propagation of a surface plasmonic mode is the dramatic slowing down of the energy flux and its consequences for the optical gain experienced by the mode, the so-called modal gain. This leads to an unusual property of the modal confinement factor^114^, which can be several orders of magnitude larger than unity, which is conventionally thought to be the upper limit in weakly guided situations. A fortunate coincidence is that, at the interface between a semiconductor and a metal, the confinement factor for the metallic region is approximately two orders of magnitude smaller than that of the semiconductor region. The former defines the modal loss, whereas the latter defines the modal gain^114,115^. This fortunate coincidence makes it possible to achieve an overall net gain, despite the fact that the material loss in metals is larger than the material gain in semiconductors. This is the reason for the existence of a giant optical gain^116^ in surface plasmonic modes, which leads to the counterintuitive but beneficial situation of a larger optical modal gain with metal than without. The plasmonic resonant slowing down of the energy propagation leads to an interesting role of the metal, namely, enhancement near the resonance: just as a metal can enhance plasmonic absorption, it can also enhance plasmonic gain. It is important to note that the definition of the confinement factor needs to be modified in a waveguide or cavity with strong confinement, such as in a plasmonic nanolaser^114,115^.

Another exciting realization is the beneficial role played by metals in small lasers despite the metal loss. The overall cavity *Q* is determined by the internal absorption and the far-field radiation loss, as given by 1/*Q* = 1/*Q*_a_ + 1/*Q*_r_, where *Q*_a_ and *Q*_r_ are the individual *Q*-factors determined by the internal absorption and far-field radiation, respectively. For a small laser, the advantage of the reduction of the far-field radiation can overcome the increased metal loss to enable an overall larger *Q* with metal^80^, or a lower threshold.

## Development of spasers and plasmonic nanolasers

### Thresholds of spasers and plasmonic nanolasers

Plasmonic devices use free electron oscillations to store electromagnetic energy and thereby can manipulate light beyond the optical diffraction limit. However, the essential field confinement capability of plasmonics is always accompanied by parasitic ohmic loss, which, in most situations, severely degrades the device performance. This led to the proposal of plasmonic amplifiers^33^. The technical challenges in realizing such devices were high. Despite widespread initial skepticism, the first reports of plasmonic lasers emerged^34–36^ 6 years later. The first semiconductor-based plasmonic nanolasers were operated at cryogenic temperatures and with high thresholds of 10–200 MW cm^−2^. The first room-temperature spaser with molecular gain had a threshold approaching 10 GW cm^−2^. In 2011, using single-crystalline semiconductor nano-squares and the total internal reflection of surface plasmons, semiconductor-based room-temperature plasmonic nanolasers were demonstrated; however, the problem of the high operation threshold of plasmonic nanolasers still was not solved. The device in ref. ^40^ was pumped by a femtosecond laser and had a threshold on the order of GW cm^−2^.

Immediately following these early demonstrations, the inherent ohmic losses and high thresholds of the demonstrated devices led to a debate about whether metals could really enhance the performance of lasers. Continuous efforts to optimize the materials and laser configuration helped to lower the thresholds of plasmonic nanolasers down to ~kW cm^−2^ at cryogenic temperatures^46,117^ and to the range of 1–100 MW cm^−2^ at room temperature^43,118^. However, these room-temperature thresholds were still 2–4 orders of magnitude higher than those of commercial laser diodes. In 2014, a concern was raised regarding whether metallic cavities could provide any advantage in laser construction at all^119^. It was pointed out that the threshold density of a spaser cannot be lowered below MW cm^−^^2^ with practically available plasmonic materials. Notably, this limit of MW cm^−2^ is approximately the threshold density for a laser with a diameter of only ~10 nm.

Despite the high losses, reasonable lasing threshold values are achieved when favorable values of both *β* and *ζ* are possible^120^. Higher *β* values emerge naturally from smaller plasmonic lasers. However, an increase of *ζ* ∝ *V*^−1^ is also possible as the volume of the gain material is reduced. For example, the smallest spasers, utilizing the molecular gain around spherical gold nanoparticles, operate with *ζ* ~ 1 and *β* ~ 1. With resonator loss rates approaching a massive 10^14^ s^−1^, these devices are possible only due to the aforementioned threshold reduction phenomena. Such spasers extend the laser phenomenon to its limits and generally require pulsed operation to avoid thermal damage to the devices.

**Fig. 4:**
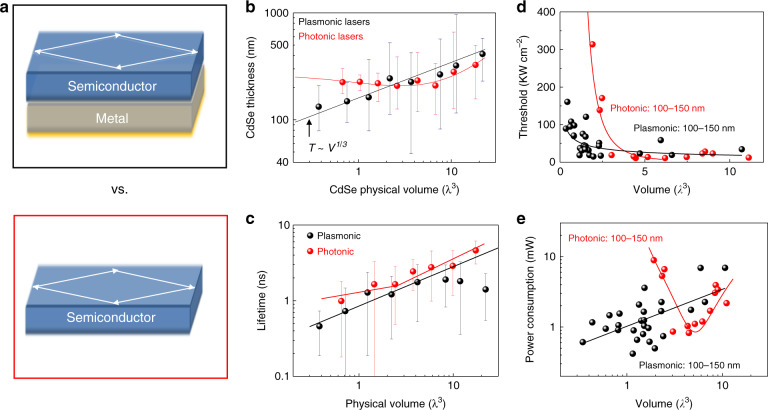
Comparison between plasmonic nanolasers and photonic nanolasers^120^. **a** In total, 170 plasmonic nanolasers (top) and photonic nanolasers (bottom) were measured for comparison, each with the same gain material and cavity feedback mechanism. **b**–**e** Comparisons of cavity size (**b**), spontaneous emission lifetime (**c**), threshold (**d**) and power consumption (**e**). These comparisons suggest that plasmonic lasers can be more compact and faster with lower power consumption than photonic nanolasers when the cavity size approaches or surpasses the diffraction limit.

In 2017, room-temperature plasmonic nanolasers with a low threshold on the order of 10 kW cm^−2^, which corresponds to a pump density in the range of modern laser diodes, were reported^120^. More importantly, in the same work, scaling laws for the key parameters of plasmonic and photonic nanolasers were reported, including the physical dimensions, threshold, power consumption, and lifetime (Fig. 4). These parameters were analyzed to identify a set of laws that dictate how these parameters scale against each other. These scaling laws suggest that plasmonic lasers can be more compact and faster with lower power consumption than photonic nanolasers when the cavity size approaches or surpasses the diffraction limit. These results clarified the long-standing debate over the viability of plasmonics in laser technology and identified situations in which plasmonic lasers have clear practical advantages. Most recently, an experiment suggested that the threshold of a plasmonic laser can be lowered to 70 W cm^−2^ via a combination of a lattice plasmonic cavity and an upconverting nanoparticle gain material^121^.

For a laser with a *β* much smaller than 1, the threshold can be easily defined as a kink in the light–light curve on a linear scale. However, this kink will vanish as β approaches 1^86^. The issue of the lasing threshold has once again become a focus of discussion with the emergence of nanolasers with *β* ~ 1. The lasing threshold has been an important issue of discussion from the very early days of lasers. The initial question was whether the threshold simply means a more dramatic increase in the laser intensity or the number of photons. It soon became clear^84^, first from a theoretical study and later through experimental demonstration that the more distinctive characteristic of the lasing threshold lies not in the laser intensity but in the photon statistics. The lasing threshold is more fundamentally characterized by a change in the equal-time intensity correlation (or second-order correlation) function *g*^2^(0) (Eq. (4)), from 2 for spontaneous emission to 1 for emission in the coherent state. Despite this clear general understanding, the issue later resurfaced, first during the development of micro-cavity lasers^122^. Plasmonic nanolasers provided an opportunity for the second revival of this debate with the fabrication of nanolasers with *β* truly approaching 1^65^. This revival prompted renewed interest in re-examining the issue of the lasing threshold. Various interesting aspects of the lasing threshold were revealed, especially for devices in which the spontaneous emission factor is close to unity^87^. It is clear now that a light-emitting device with a unity spontaneous emission factor does not become a laser at zero pumping. The absence of an intensity kink does not imply a threshold of zero for a nanolaser, as we discussed earlier in this paper. Finite pumping is always required even when the spontaneous emission factor is unity. In fact, the transition in *g*^2^(0) from larger than 1 to 1 becomes much softer, and it may take very high pumping for *g*^2^(0) to eventually approach 1 when *β* = 1. An LED gradually becomes more like a laser with increased pumping. The sharp threshold seen in a larger laser now becomes a range of pumping values in which *g*^2^(0) slowly approaches unity. The device may be thresholdless in the sense of the absence of a sharp kink, but it definitely does not become a laser at zero pumping. This scenario raises an interesting question: what is the quantitative definition of a laser, or how close to 1 must *g*^2^(0) be for a device to qualify as a laser? More complete and up-to-date discussions of the threshold issue for high-*β* micro- and nanolasers have been presented from both theoretical and experimental perspectives by Chow and Reitzenstein^123^.

### Electrically injected nanolasers

Optical pumping has played an important role in proof-of-concept studies and in revealing basic physical effects. However, for certain applications, electrical injection is necessary, especially for semiconductor-based nanolasers with intended applications in integrated photonic circuits. Electrical injection in plasmonic nanolasers presents both interesting opportunities and challenges^80^. It is both convenient and practical to use the same metal both for plasmonic confinement and as the contacts for electrical injection, as in the original design^70,124–126^. The challenges involve the increased degree of difficulty in the fabrication of high-quality metallic structures with small features. There are also intrinsic issues related to the increased contact resistance for smaller structures^127,128^. For electrically injected plasmonic lasers, approaches involving traditional III–V fabrication techniques seem to be more feasible^27,34,70,124–126^.

**Fig. 5:**
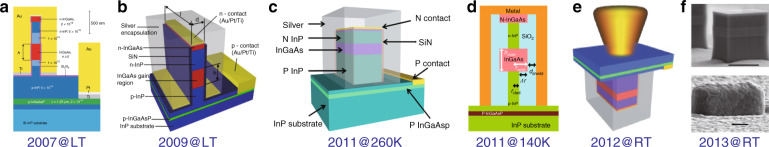
Development of electrically injected plasmonic and metallic cavity nanolasers. LT: low temperature; RT: room temperature. **a** First electrically injected metallic cavity nanolaser operating in dielectric mode^27^. **b** First electrically injected nanolaser operating in metaldielectric-metal plasmonic gap mode^34^. **c**, **d** Nanolasers operating above liquid nitrogen temperature^129,130^. **e**, **f** Demonstrations of the first room-temperature operation of electrically injected metallic cavity nanolasers^66,125^. Notice that except in **b**, all of these metallic cavity lasers operate in dielectric modes.

Figure 5 presents major progress in electrically injected metallic cavity nanolasers, from the first demonstration of a metal-encased cavity operating in dielectric mode (Fig. 5a) and the first operation in plasmonic gap mode (Fig. 5b) to subsequent efforts to increase the operation temperature of electrically injected nanolasers. It is important to note that all but one (Fig. 5b) of these lasers work in dielectric modes. More extensive research and optimization are needed for device configurations for electrical injection beyond the current approaches^8,65,70,80,124–126,129,130^. Another challenge for electrical injection for eventual high-speed applications is to minimize the RC delay, which will require careful design to avoid the occurrence of large capacitance due to the plasmonic or contact metals.

### Towards room-temperature operation

Room-temperature operation is a requirement for many practical applications and is typically a milestone in the development of any new type of laser. For plasmonic nanolasers, room-temperature operation represents an even larger challenge due to the severe plasmonic heating. It was completely unclear from the outset whether plasmonic nanolasers would ever be able to operate at room temperature. One of the earliest demonstrations of room-temperature operation was presented by Ma et al.^40^ using a semiconductor nano-square coupled with silver film under optical pumping. For a device operating under electrical injection, it was a much longer process from the initial demonstration at 10 K^24^ to 260 K^129^ and, eventually, to room temperature^66,125^. It is important to note that room-temperature operation under electrical injection has been demonstrated for dielectric modes, not for plasmonic modes. Therefore, strictly speaking, plasmonic nanolasers operating under electrical injection have not yet been realized at room temperature. Both innovative designs and systematic efforts will be needed to achieve lasing in plasmonic modes under electrical injection at room temperature.

### External quantum efficiency of plasmonic nanolasers

The inevitable metallic absorption loss in plasmonic nanolasers converts the input power into heat rather than radiation, leading to an undesired low external quantum efficiency (EQE) and device degradation. Any characterization of the quantum efficiency of a plasmonic nanolaser should consider its near-field surface plasmon emissions, divergent emission profile, and limited emission power. In 2018, a method of characterizing the external quantum efficiency of plasmonic nanolasers was developed by combining experimental measurements and theoretical calculations^131^. Through systematic device optimization, high-performance plasmonic nanolasers with an external quantum efficiency exceeding 10% were demonstrated at room temperature. The EQE of plasmonic nanolasers can be further increased by accelerating their radiation rate by employing a smaller cavity with a lower radiation quality factor, for which optimizations in terms of cavity configuration and metal quality are crucial. A higher EQE can also be realized by coupling a plasmonic nanolaser to an embedded or adjacently integrated waveguide^41,126^. Through waveguide coupling, the emission directionality of plasmonic nanolasers can also be recovered^41^.

### Dark emission of plasmonic nanolasers

**Fig. 6:**
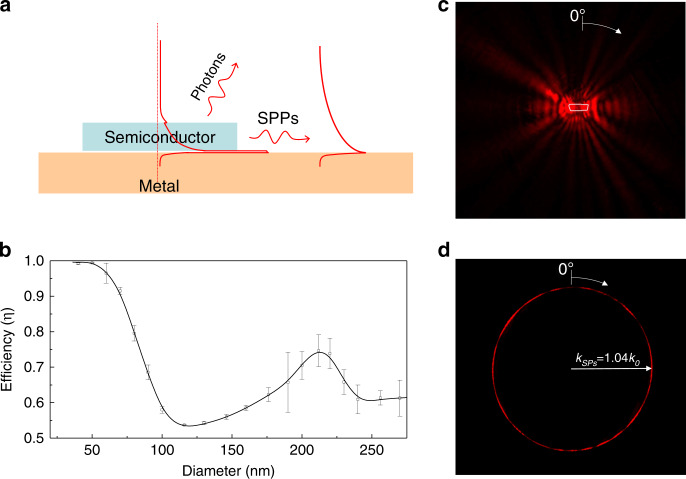
Dark emission of plasmonic nanolasers^132^. **a** Schematic of the radiation of a plasmonic nanolaser based on a metal-insulator-semiconductor gap mode, which consists of free-space photon radiation and SPP dark emission. **b** Surface plasmon generation efficiencies of nanowire plasmonic nanolasers with various diameters operating in the fundamental plasmonic mode. **c**, **d** Real-space (**c**) and momentum-space (**d**) images of plasmonic nanolaser emission obtained via leakage radiation microscopy. The white polygon in **c** indicates the profile of the nanolaser.

In contrast to conventional lasers, a plasmonic nanolaser (spaser) amplifies surface plasmons instead of propagating photons, providing amplification of light localized at a scale smaller than the diffraction limit. Therefore, the surface plasmon “emission” of a plasmonic nanolaser is considered to be “dark”, if it does not couple to the far-field (Fig. 6). In 2017, the surface plasmon emission of plasmonic nanolasers was directly and simultaneously imaged in the spatial, momentum, and frequency spaces using leakage radiation microscopy^132^. The results showed that a plasmonic nanolaser can serve as a pure surface plasmon generator, with nearly 100% of its emission radiating into the propagating surface plasmon mode outside of the cavity. Experimentally, a nanowire plasmonic nanolaser was fabricated, and ~74% of its total emission radiated into the propagating surface plasmon mode outside of the cavity (Fig. 6).

### Eigenmode engineering of spasers and plasmonic nanolasers

The eigenmode of a nanolaser with a high *β* can be engineered in a controllable manner for novel inner laser cavity field and/or emission beam synthesis. Recently, a new class of lasers based on concepts borrowed from parity-time symmetry in quantum mechanics has emerged^133^. A recent study indicates that a parity-time-symmetric synthetic plasmonic nanocavity can be employed to construct a vortex nanolaser^134^. When a single dipole emitter interacts with such a nanocavity, a counterintuitive phenomenon emerges in which the radiation field of the dipole can display the opposite handedness to the coalesced eigenstate of the system at an exceptional point, which violates the conventional wisdom that an emitter radiates into and interacts with eigenstates of the photonic environment^134^. Furthermore, ensembles of nanolasers operating in unison can produce a macroscopic response that would not be possible in conventional lasers. In this case, it is the structure at the nanoscale that determines a laser’s operational characteristics, in a manner similar to a metamaterial. In the near-field, the polarization and profile of each nanolaser eigenmode can be controlled, with additional control over the ensemble through coupling, relative phase, eigenmode symmetry and topology. Such cooperative eigenmode engineering is distinct from that for photonic crystal lasers, for which periodicity is the main control parameter, and could enable unprecedented control of macroscopic laser fields, with a range of far-field applications^135–137^.

### Single-nanoparticle spasers

**Fig. 7:**
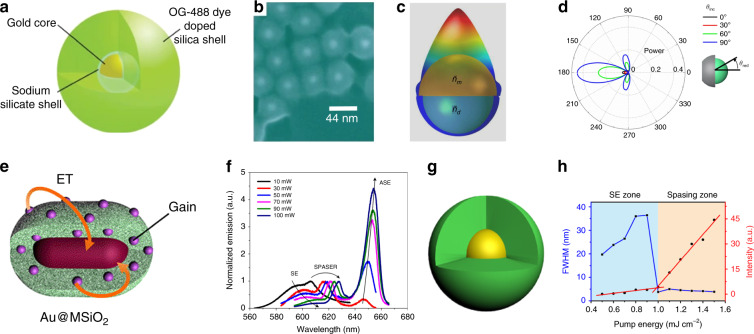
Single-particle spasers. **a** A schematic of a single-particle spaser design involving a gold core covered by a silica shell doped with an organic dye (Oregon Green 488)^36^. **b** Scanning electron microscope image of spaser nanoparticles^36^. **c** Radiation from a metal-semishell-capped spaser. The silver semishell generates feedback with localized surface plasmons, whereas the porous silica core provides optical gain^138^. **d** A polar plot of the power flow from the spaser featured in **c** at different angles of incidence^138^. **e** A spaser design consisting of a gold nanorod covered with an organic dye-gain material embedded in a mesoporous silica shell^139^. **f** The spontaneous emission (SE) and amplified spontaneous emission (ASE) from the spaser system in **e** with different dye concentrations. Each spectrum is normalized to the peak of the spaser emission^139^. **g** A schematic of a core-shell spaser with a gold core covered by a three-level dye system embedded in a silica shell^140^. **h** The evolution of the full width at half maximum (FWHM) (blue) and emission intensity (red) of the spaser in **g** vs. the pump energy^140^.

A single-particle plasmonic nanocavity is of immense interest due to the potential to reach extremely small mode volumes and ultrahigh Purcell factors. Immersing a single nanoparticle in a gain medium can lead to the smallest laser device, a spaser. Inspired by this concept^33^, many groups have presented experimental demonstrations of spasing action, including the use of plasmonic nanoparticles^36^ as well as waveguides^34,35^. The first report on single-particle spasers demonstrated a gold nanoparticle core serving as the feedback plasmon nanocavity coated with a shell of organic dye-doped silica^36^. Figure 7a depicts this ultracompact spasing system, with a gold-core diameter of 14 nm and a shell diameter of 44 nm, as further detailed in Fig. 7b. This original experiment has been followed by others with an eye towards improving the design and addressing the initial limitations, such as a lack of directionality^138^, a lack of spaser wavelength tuneability^139^, and the need for a high threshold to overcome the material losses in the visible region^140^. Accordingly, directionality in single-particle spasers has been achieved by breaking the symmetry of the core-shell structure^138^ (Fig. 7c). Utilizing a metal-semishell-capped spaser instead of a closed metal shell design enabled the spasing emission to be directed towards a preferred direction (Fig. 7d) independently of the polarization and incidence angle of the pump. This metal-semishell resonator spaser can also improve the power efficiency of the original design by minimizing undesired radiation. Figure 7e depicts a different spaser structure used to achieve wavelength tuneability^139^. This structure consists of a gold nanorod coated with a monolayer of mesoporous silica as a single-particle plasmonic nanocavity and organic laser dyes as optical gain media. This design provides more efficient energy transfer from the gain material to the gold nanorods due to the ability to encapsulate the optical gain inclusions inside the mesoscopic pores of the silica shell. Figure 7f demonstrates that by adjusting the doping level of the dye, the spaser emission wavelength can be tuned from 562 to 627 nm. Other limitations, such as the high threshold required to compensate for material loss and the short lasing lifetime, have been addressed through the engineering of the triplet state in a three-level system^140^. This three-level spaser utilizes a structure very similar to that of the original single-particle spaser, as shown in Fig. 7g. In addition, the spasing threshold and dynamics in the new spaser can be tuned by engineering the energy levels of the gain medium and the energy transfer process. The three-level spaser demonstrates a low threshold of 1 mJ cm^−2^ (Fig. 7h). The mean-field chromophore interaction is included through a field- and position-dependent dielectric function with gain saturation. However, more complex cooperative effects^141,142^ have been intentionally omitted, as they should be smeared out by strong dephasing and random dipole orientations. A recent study^143^ has confirmed that for tens of thousands of randomly oriented molecules, the ensemble-averaged dipole–dipole coupling should vanish, and the resonant mode should remain unaffected.

### Lasing from periodic spaser arrays

**Fig. 8:**
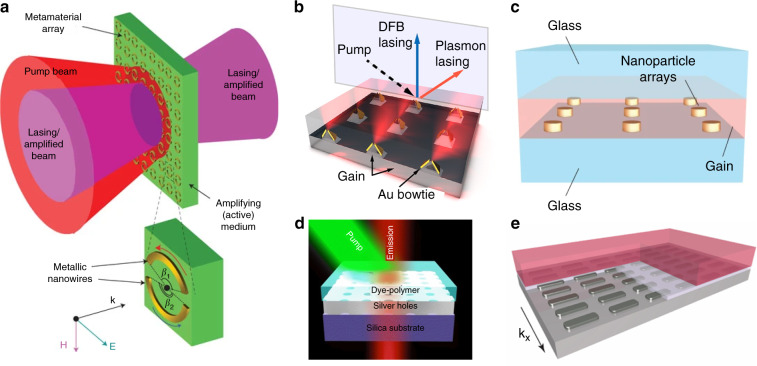
Spasers consisting of arrays of plasmonic nanoparticles. **a** Proposal of a two-dimensional arrangement of resonators to achieve a directional spaser array^144^. **b** A plasmonic array of bowtie nanoantennas covered with an organic gain material^54^. **c** Strongly coupled plasmonic nanocavity array^74^. **d** Periodic nanohole spaser array covered with an organic dye-gain medium^146^. **e** Low-threshold plasmon–exciton–polariton spaser obtained with an array of silver nanoparticles^151^.

Some of the limitations encountered with single-particle spasers are inherent to their design, and therefore, a different spaser system would be required to overcome these challenges. A single-particle spaser has a single source of feedback, which limits the quality factor of the resonator. The structural engineering of arrays of plasmonic resonators has been applied to address the high radiative losses and poor directionality of spasers^54,74,75,144–146^. It was proposed in 2008 that the arrangement of plasmonic particles in a two-dimensional array would lead to interactions between the individual plasmonic elements, giving rise to a high-quality-factor collective SPP resonance^144^. This type of quantum generator is called a lasing spaser^144,147^. Such a spaser is a periodic array of individual spasers that interact in the near-field to form a coherent collective mode. Such lasing spasers are built of plasmonic crystals that incorporate gain media. One type of lasing spaser is a periodic array of holes in a plasmonic metal film deposited on a semiconductor gain medium^75^, and another type is a periodic array of metal nanoparticles surrounded by a solution of dye molecules^74^. Once such an array is brought in contact with a gain medium, the radiation losses and Joule losses in the metal can be compensated, and above a certain gain threshold, the array will begin spasing. In the plane of the array, the strongly trapped current modes in the plasmonic resonators oscillate in phase, leading to spasing emission in the normal direction, as depicted in Fig. 8a. A critical step in the experimental realization of spaser arrays was the incorporation of a gain material into the high-local-field areas of a negative-index metamaterial (NIM). That localized embedding of the gain material enabled the experimental demonstration of an extraordinarily low-loss active optical NIM in the visible wavelength range between 722 and 738 nm. With loss compensation, the original loss-limited negative refractive index was drastically improved. Thus, at a wavelength of 737 nm, the negative refractive index was enhanced from −0.66 to −1.017^145^.

Further experimental demonstrations followed, showcasing spasing action in arrays of coupled plasmonic nanoparticles^54,74^ (Fig. 8b, c) and nanoholes in plasmonic films^75,146^ (Fig. 8d). As predicted, such spaser nanoparticle and nanohole arrays have demonstrated directional emission and lower thresholds than single-particle spasers due to the higher quality factors of the resonances. Superlattices of plasmonic arrays have been structured to access multiple band-edge modes, leading to multi-modal lasing at arbitrary wavelengths^77^. Other exciting developments in spaser arrays have included new material platforms. For example, a high-loss ferromagnetic plasmonic material has been employed for tuneable multi-modal lasing spaser arrays^148^, whereas broadband lasing feedback has been achieved using a hyperbolic metamaterial relying on a large photonic density of states^149^. In addition, the tuneability of the spasing wavelength using a stretchable spaser array for mechanical modulation of the spaser feedback has been demonstrated^78^, as well as real-time tuning of the spaser wavelength through modification of the refractive index of the gain material^150^. The spaser concept has recently been extended to polariton lasers through the strong coupling between dye excitons and plasmonic modes in a plasmonic array (Fig. 8e). The resulting plasmon–exciton-polariton spaser array exhibits laser-like emission at a significantly lower pump threshold than in conventional spasers^151^.

A recently proposed topological lasing spaser was built of a honeycomb plasmonic crystal consisting of silver nanoshells with a gain medium inside^135^. The generating modes of such a spaser are LSPs with chiral topological charges of *m* = ±1. This difference in the charges topologically protects them against mixing: only one of the *m* = ±1 topologically charged modes can be generated at a time, as selected by the spontaneous breaking of time-reversal (${\cal{T}}$) symmetry.

We anticipate that spasers will have a central role in future on-chip integrated technologies due to their ability to generate coherent fields at the nanoscale. Future developments of spasers will include electrically driven spasers that can out-couple their emission into the modes of plasmonic waveguides. In addition, the optimization of spasers compatible with CMOS materials will be essential for their adoption in more applications as well as their commercialization.

### Applications of single-particle spasers

The exceptional properties of single-particle spasers are potentially crucial to a large number of applications, especially sensing and imaging. Single-particle spasers have great advantages as luminescent probes^38^. Spasers can serve as ultrabright biocompatible biological probes. The ability to produce stimulated emission directly inside living cells has been achieved^38^ with a gold-core silica shell single-particle spaser, acting as a much brighter probe than conventional ones. The ability to reduce the spasing threshold and increase the spasing lifetime would make spasers highly efficient advanced luminescent probes. Among the applications that have been demonstrated as proofs of principle, we mention applications in sensing and detection in particular^42,44,71^, which are fundamentally based on the abovementioned fact that the spasing frequency depends on the geometry and composition of the spaser (Eq. (2)) and rather weakly, if at all, on the pumping and ambient temperature. A shift in a spaser indicates a change in the nano-environment close to the spaser due to the presence of the analyte. It has been shown^42,44,71^ that nanolasers built of nanorods over metal are very sensitive to minute concentrations of analytes, allowing for unprecedented detection sensitivity.

**Fig. 9:**
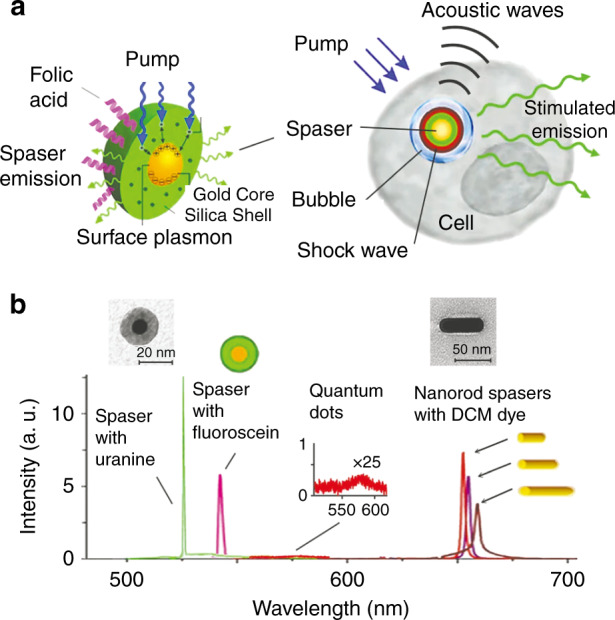
Structure and spectra of spasers. **a** Left: Schematic of nanoshell spaser and its function inside a living cell. The spaser consists of a gold nanosphere core surrounded by a porous silica shell impregnated with a dye (uranine). Right: Schematic of a cell and the processes accompanying pulse spasing. A pump laser pulse causes spaser generation; where stimulated optical emission occurs, a vapor bubble is formed due to heat production, and a shock wave and acoustic waves are launched. **b** Spectra of investigated spasers: a nanoshell with uranine dye, a nanoshell with fluorescein dye, and three gold nanorods surrounded by shells with DCM dye. For comparison, the spectrum of quantum-dot radiation is shown magnified by ×25. Electron micrographs of a 20-nm spherical spaser and a 50-nm nanorod spaser are also shown at the top. Adapted from ref. ^38^.

Importantly, true nanospasers, which are nanometric in all dimensions, have the distinct advantage of being of the same order of magnitude in size as biomolecules and transistors. This attribute opens up unique and important avenues of application. One demonstrated application is in cancer therapeutics and diagnostics (theragnostics)^38^. To briefly discuss this application, we begin with Fig. 9, in which the spectra and geometry of the studied nanospasers are displayed. As is characteristic of spasing, the spaser emission spectra are extremely narrow, ~1 nm in width. The spaser used in the cancer theragnostics experiments was the 20-nm gold-core nanoshell spaser. The surface was functionalized to make it adhere to the surfaces of cancer cells that exhibit a large concentration of folate. The narrow spectrum and very high spectral intensity of the spaser emission enabled unprecedented confidence in the detection of cancer cells.

**Fig. 10:**
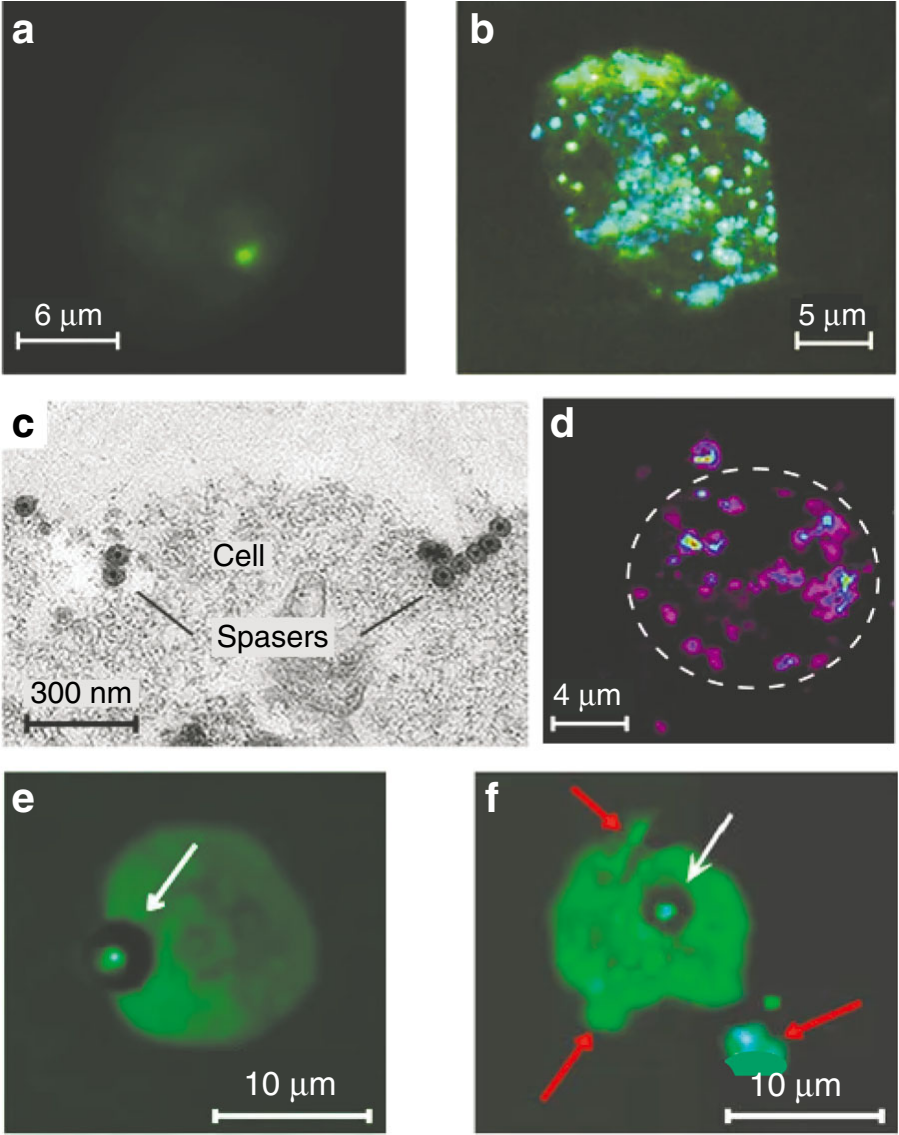
Spasers inside a cancer cell. **a** Optical micrograph of a single spaser radiating inside a cell. **b** Optical micrograph of multiple spasers generating radiation inside a cancer cell. **c** Electron micrograph (positive contrast) of multiple spasers inside a cell. A single spaser, a dimer of spasers, and a cluster of multiple spasers are clearly seen. **d** Photothermal image of a cell labeled with multiple spasers. **e** Optical micrograph showing a single spaser (marked with a white arrow) producing a surrounding vapor bubble. **f** Optical micrograph illustrating a mechanism of cancer therapeutics using spasers. A single spaser surrounded by a vapor bubble is indicated by a white arrow. Red arrows indicate fragments of the cell membrane due to damage caused by shock waves produced by the cavitation around the spaser. The cancer cell dies after one or a few laser pulses. Adapted from ref. ^38^.

The theragnostic use of the spaser is illustrated in Fig. 10. Living cancer cells were incubated with spasers whose surfaces were functionalized to bind with these cells. During this process, the cancer cells absorbed the spasers, which generated radiation inside the cells when pumped. Different numbers of spasers per cell can be internalized in this way depending on their concentration and the incubation time: from a single spaser, as in Fig. 10a, to a multitude of spasers, as in Fig. 10b. Spasers inside a cell as visualized by electron microscopy are shown in Fig. 10c: one can see an isolated spaser, a dimer of spasers, and an aggregate of many spasers. Note that a spaser makes an excellent fluorescent label because it is literally brighter than a million quantum dots! Spasers also makes excellent agents for photothermal imaging (Fig. 10d) because of their very high absorption cross section.

The therapeutic effect of spasers is illustrated in Fig. 10e, where with an increase in pumping, the formation of a vapor bubble around the spaser, produced by the heat released into the surrounding liquid, is clearly seen. The damage to cancer cells that is produced by spasers pumped at such levels is demonstrated in Fig. 10f, where the cell membrane is ruptured at several sites and membrane fragments can be clearly seen. This leads to the death of a cancer cell after one or several laser pulses. The mechanism of this damage is certainly not thermal: the volume of the spaser is ~10^3^ nm^3^, while the volume of the cell is ~10^3^ µm^3^, i.e., larger by a factor of a million, which suggests that the increase in the mean temperature of the cell during a pulse is negligible.

The exceptional efficiency of spasers is not incidental: it is based on their fundamental photophysics as unsaturable absorbers and emitters. In spasers operating at a sufficiently high pump intensity, the SP emission is predominantly stimulated in nature, meaning that the SP population is proportional to the pumping intensity. Therefore, saturation is absent, and any deviations from a straight L–L line are due only to changes in the constituent materials. Over a very wide range of pumping intensities, spasers exhibit a linear L–L line and unsaturable behavior, causing them to show unprecedented brightness as fluorescent labels, extremely narrow spectral emission lines, and high efficiency as photothermal and photoacoustic agents^38^.

**Fig. 11:**
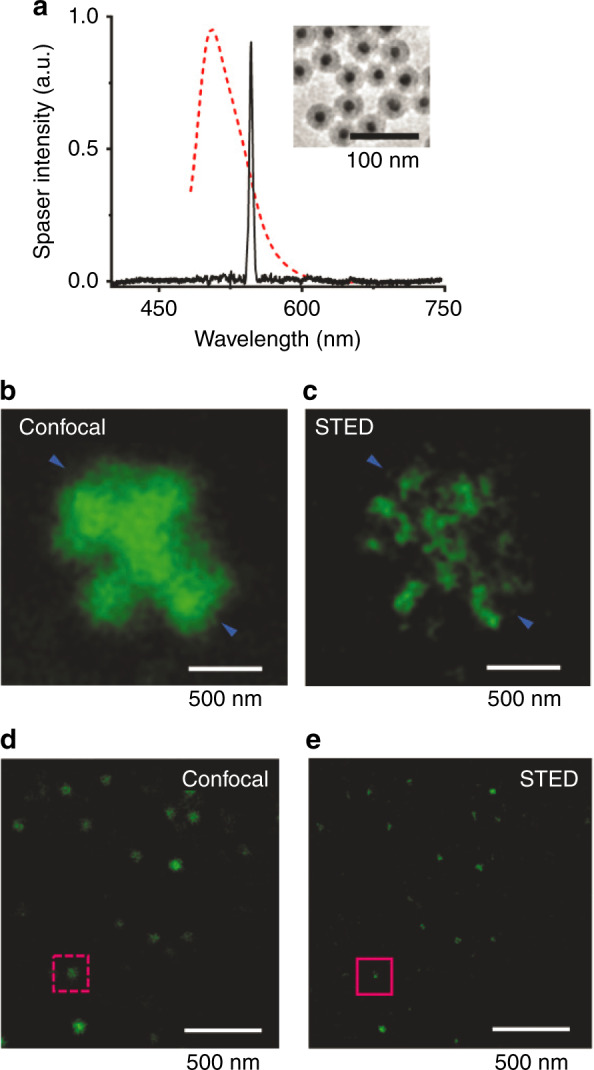
Use of spasers for stimulated emission depletion (STED) super-resolution imaging. **a** Emission spectrum of the gain-medium dye (a fluorescein derivative) (red dashed line) and the spasing spectrum (solid black peak). An electron micrograph of the spasers is shown in the inset. **b** Optical image of radiating spaser aggregates under a confocal microscope. **c** The same as in **b** but obtained using STED super-resolution microscopy. **d** Confocal optical image of separated single spasers. **e** The same as in **d** but obtained using STED super-resolution microscopy. Adapted from ref. ^39^.

A proof-of-principle demonstration of spasers as efficient agents for stimulated emission depletion (STED) super-resolution microscopy has been published recently^39^ (Fig. 11). STED super-resolution microscopy^152,153^ is applicable to objects labeled with fluorescent dyes. Two optical pulses are applied: (i) a pumping pulse that causes population inversion, focused as close to the diffraction limit as possible, and (ii) a depletion pulse applied at a fluorescence frequency that causes stimulated emission and depletes the population inversion. This depletion pulse carries optical angular momentum and, consequently, has a donut shape in the focal plane. Due to the nonlinear nature of its interaction with the dye, the un-depleted area at the center of the “donut” is significantly smaller than the wavelength, enabling super-resolution.

This idea of STED is fully applicable not only to spontaneously emitting dye molecules but also to spasers, in which the second pulse depletes the population inversion and stops the spasing^39^. The spaser used in ref. ^140^. exhibits an extremely narrow spectrum of generation, as shown in Fig. 11a. The STED super-resolution imaging achieved using these spasers is remarkable, as is evident from comparing confocal images of spaser-nanoparticle aggregates (Fig. 11b) with images of the same aggregates obtained using STED (Fig. 11c). The achievement of super-resolution is even more evident from a comparison of confocal images of isolated single spasers (Fig. 11b) with corresponding STED images of the same spasers. These observations show the high promise of spaser-based STED far-field nanoscopy for applications in the biomedical field and others.

### Multiphysics analysis of spasers with experiment-based gain models

The new, exciting experimental studies of spasers and SPP nanolasers over the last decade have been supported by computational efforts developed to design and analyze critical performance metrics obtained under real-world conditions and other vital details of operation^140,154–162^. Thus, power balance and heating have been studied to guide spaser design in terms of the allowed pumping intensities, duration, and expected output radiation and heating of the devices^163^. In addition, the gain threshold requirements for metallic-dielectric core-shell single-particle spasers have been theoretically derived considering the interband transitions of the metal^155^. These studies have shed essential light on the parameters affecting the threshold, including the resonant wavelength, the refractive index of the background host material, and the dimensions of the core and shell of the spaser. Classical electrodynamical models in which the quantum-mechanical effects of the gain medium are considered by introducing nonlinear permittivity have been initially adopted to describe spasing^156,157^. Such models are capable of adequately predicting the conditions for loss compensation and the transition to the spasing regime for simplified geometries and operation regimes. However, as the designs of spaser systems are becoming more involved, full-wave numerical analyses that can unlock the temporal and spatial details of a given spaser are required. Time-domain multiphysics techniques are considered amongst the most accurate approaches for accounting for the quantum-mechanical nature of the gain and plasmonic materials, as they naturally combine nonlinear and thermal effects in computational domains with complex geometries^158–162,164,165^. In the time-domain multiphysics approach, the Maxwell equations are coupled to a multi-level model of the gain material through auxiliary differential equations (ADEs). Such multi-level atomic models enable the integration of the rich physics of the light-matter interactions in the gain materials of spasers and SPP nanolasers into time-domain schemes, allowing the electromagnetic field and carrier population in any time step to be calculated under the influence of different pump intensities^158^. Such models naturally incorporate diverse effects such as gain saturation and radiative and nonradiative transitions taking place in the active medium.

Further developments of the early multi-level models have included the incorporation of pumping dynamics or the Pauli exclusion principle^159^, the photobleaching of a laser dye^161^, quantum noise^160,162^, thermal management, and other intricate phenomena. It has been shown that the experiment-based kinetic parameters of gain media enable high predictive power of such multi-level atomic models for designing spasers and SPP nanolasers^165^.

## Future perspectives on nanolasers and spasers

### Ultimate miniaturization

One of the most appealing aspects of plasmonic nanolasers and spasers is the significant size reduction they offer, far beyond what is possible with pure dielectric or semiconductor laser structures. Such lasers represent the first-ever opportunity to finally make lasers with sizes compatible with electronic devices. The spasers demonstrated originally had sizes of ~40 nm in diameter. Such spasers are often prepared in solution and are ideal for applications in solution-based sensing and detection. For other applications intended for information transmission in integrated photonics^8,166^, devices need to be fabricated on solid substrates and operated under electrical injection. Such devices are often much larger, especially for rectangular devices in which electrical injection structures are included. Devices that work at room temperature in CW mode have sizes on the order of wavelengths in vacuum^8,80,166^. Design and simulation studies have shown^66,126^ that a multi-layer structure based on traditional III–V semiconductors and fabrication technology could lead to SPP lasers as small as ten-thousandths of a wavelength cubed in vacuum. Such designs can be realized using traditional semiconductor wafers combined with the membrane transfer technique, as demonstrated recently^167^. Two recent demonstrations^168,169^ of the effective coupling of single emitters with plasmonic bowtie structures or between a metallic sphere and a surface raise an interesting question regarding the ultimate size of a laser. In both cases, strong coupling and SPPs were observed with a Rabi splitting as large as 300 meV. It would be extremely interesting to see whether such a structure could be driven into the lasing regime and would thus represent the ultimate size miniaturization of lasers. Room-temperature operation of such devices under electrical injection would be even more exciting but may currently present significant challenges.

Poor metal quality remains one of the most severe challenges for fabricating small nanolasers with a low threshold and high efficiency. The ideal long-term solution would be the all-epitaxial growth of semiconductors together with plasmonic structures^168^ (in the form of highly doped semiconductors or metals) for plasmonic confinement and contact. Such an approach would lead to simplified fabrication and high quality of the overall device structures. Another potentially promising approach is related to the 2D flat materials that are currently emerging^170^, including semiconductor gain materials and metallic materials. These 2D flat materials are ideal for plasmonic coupling with controlled separation between the gain and metallic layers, offering single-crystal quality of 2D metals. Plasmonic coupling and enhanced 2D emission have been demonstrated^171–174^, as well as optical gain^175,176^ and lasing based on such 2D materials^177–179^ using regular nanophotonic cavities. Integrated SPP laser structures based entirely on 2D materials, including 2D semiconductor, dielectric, and metallic layers, could potentially lead to the smallest possible plasmonic lasers, with many advantages.

### Miniaturization of photon condensates

To decrease the threshold and power consumption, smaller devices with only a few optical modes are highly desirable. Under these conditions, spontaneous and stimulated emission are restricted to a small subset of modes that couple strongly to the gain material. In the case of strong coupling, this can lead to hybrid states of light and matter known as polaritons^180^, as previously discussed. As these hybrid exciton states strongly interact with each other, they can attain near-thermal equilibrium conditions; thus, under stimulated scattering, polaritons may condense, akin to the formation of a Bose–Einstein condensate (BEC)^181–184^. The emergence of thermal equilibrium in the stimulated emission process enables an additional degree of control over the optical modes.

Even in the weak coupling regime between light and matter, thermal equilibrium is also achievable^185–190^. This phenomenon occurs naturally in optical cavities where *ζ* ≤ 1: this naturally promotes photon absorption and re-emission, and via the gain material’s phonons, the light within the cavity can attain thermal equilibrium^191^. When the light within the cavity is driven to a critical photon number, a laser-like response emerges in the lowest energetic mode that overlaps with the spectral response of the gain material. It has been shown that this response closely follows a Bose–Einstein distribution, with the laser transition being analogous to the condensation of the cavity photons.

Even though such analogies between laser action and BECs are compelling, their primary utility lies in the ability to employ the highly successful machinery of thermodynamics and statistical mechanics to understand the interactions of laser modes^66,126^. The two fields have much to offer each other: statistical mechanics can be used to describe complex mode competition in lasers, while photonic BECs can be produced far more rapidly than atomic BECs and at room temperature. One exciting possibility stems from reducing the size of a photonic BEC: just as the threshold of a nanolaser is reduced through miniaturization, so too is that of a photonic BEC. In terms of statistical mechanics, this also results in a reduction of the number of photons within the BEC. To date, a photon BEC has been demonstrated with just 7 photons^189^.

### Nano-LEDs and spontaneous emission control

Conventional macroscopic optical cavities require stimulated emission of the required optical modes if they are to be isolated from unwanted modes. Indeed, for lasers larger than the wavelength of the light, this is the only means by which such mode control is possible, as all modes share spontaneous emission approximately equally. However, nanocavities can exploit the Purcell effect, completely changing the equal distribution of spontaneous emission among the modes. Indeed, this is the method by which nanocavities may achieve *β* → 1. This raises the question of whether stimulated emission is required at all. Some may argue that lasers are faster than LEDs, but the Purcell effect improves the situation here as well. In fact, nano-LEDs may well be just as effective as nanolasers from an energy conversion point of view^192^. However, nano-LEDs and nanolasers will still have different photon statistics and noise properties^69^. For applications in which noise is not an issue, nano-LEDs may suffice. Another issue of concern for nano-LEDs could be limited emission power due to the lower excitation level of the gain materials.

### Spaser-based interconnects

Finally, one of the most promising applications of spasers is their novel use for on-chip optical interconnects^99^, beyond what has already been discussed for conventional optical interconnects^8^ This proposed application, which has not yet been demonstrated experimentally, can resolve the most important problems of electronic information processing: the limited clock rate of a processor (realistically, not exceeding several GHz) and high heat production. Both of these drawbacks originate from the same fundamental physics: the coupling between the transistors on a processor chip is electrostatic. When a transistor flops its state, the interconnect must be recharged by the current from a single transistor, which requires a long time and releases electrostatic energy as heat. However, a fundamentally different principle has been formulated^99^ based on the use of SPPs to bring a signal from one transistor to another. In this case, a transmitting transistor electrically pumps a spaser that has the same ~10 nm size as the transistor. The spaser is loaded by an SPP waveguide (for this purpose, the same type of copper interconnect as is used for the current electrical interconnects can be applied). On the other side, the SPP pulse is converted into a charge by a germanium nanocrystal and fed to the receiving transistor. It has been shown^193^ that a single nanoscale transistor produces sufficient drive current to electrically pump a spaser. Thus, this principle of spaser-mediated SPP interconnects is fundamentally realistic and, indeed, very promising.

## Summary

In this paper, we have attempted to present an overview of the field of nanolasers and spasers over the last 10 years, since the first experimental demonstrations in 2009^34–36^. The paper started with a brief historical overview of laser miniaturization, which eventually led to the design and first demonstrations of plasmonic nanolasers and spasers. This was followed by a summary of the fundamental properties of spasers. An overview of physics- and technology-driven motivations for small lasers was then presented; these motivations include developing a nanoscale coherent source, controlling and reducing the lasing threshold, accelerating the time dynamics of lasers for application in information transmission, and the loss-gain trade-off. The significant progress achieved in the development of spasers and nanolasers over the last 10 years was then described. The critical advancements include continuous threshold reduction, electrically injected operation, raising the operating temperature, improving quantum efficiency, progress in the capabilities of the originally developed single-particle spasers, the capability of array operation of plasmonic emitters, and multiphysics modeling and simulation. The final part of this paper presented our perspectives on the field, highlighting several important future directions of fundamental and applied research. These directions include addressing the critical question of the ultimate dimensions of a plasmonic nanolaser, both in principle and in practice, especially considering the possibility of electric injection; a new possibility of miniaturizing Bose–Einstein condensates; controlling spontaneous vs. stimulated emission; and finally, the relative merits of lasers at the ultimate small scales. Some of these and related unresolved issues and future perspectives have also recently been presented elsewhere^8^. We believe that future research towards achieving these goals and resolving these fundamental problems will further deepen our comprehensive understanding of the physics of interactions between photons, plasmons, and matter and broaden the applications of plasmonic nanolasers and spasers.
